# Adherence to social distancing measures in southern Brazil, 2020/2021: a cross-sectional study

**DOI:** 10.31744/einstein_journal/2024AO0223

**Published:** 2024-06-13

**Authors:** Pâmela Moraes Volz, Vanise dos Santos Ferreira Viero, Elizabet Saes-Silva, Bianca Languer Vargas, Fernanda Oliveira Meller, Antônio Augusto Schäfer, Simone dos Santos Paludo, Lauro Miranda Demenech, Lucas Neiva-Silva, Samuel Carvalho Dumith

**Affiliations:** 1 Universidade Federal do Rio Grande Rio Grande do Sul RS Brazil Postgraduate Program in Health Sciences, Universidade Federal do Rio Grande, Rio Grande do Sul, RS, Brazil.; 2 Universidade Comunitária da Região de Chapecó Chapecó SC Brazil Universidade Comunitária da Região de Chapecó, Chapecó, SC, Brazil.; 3 Universidade do Extremo Sul Catarinense Criciúma SC Brazil Postgraduate Program in Public Health, Universidade do Extremo Sul Catarinense, Criciúma, SC, Brazil.; 4 Universidade Federal do Rio Grande Instituto de Ciências Humanas e da Informação Rio Grande do Sul RS Brazil Instituto de Ciências Humanas e da Informação, Universidade Federal do Rio Grande, Rio Grande do Sul, RS, Brazil.; 5 Universidade Federal do Rio Grande Center for Psychological Studies Rio Grande do Sul RS Brazil Center for Psychological Studies, Universidade Federal do Rio Grande, Rio Grande do Sul, RS, Brazil.

**Keywords:** Physical distancing, Behavior, COVID-19, Coronavirus infections, Pandemics, Public health surveillance

## Abstract

**Objective:**

We aimed to analyze the frequency of adherence to social distancing and individual protection measures of adult and older populations in two cities in southern Brazil and to characterize the sociodemographic aspects of these individuals.

**Methods:**

This cross-sectional, population-based study was conducted in the cities of Rio Grande, RS, and Criciúma, SC, Brazil. The outcome measure was the frequency of adherence to social distancing measures. Fisher's exact test (5% significance) was used to calculate the differences in prevalence according to exposure variables.

**Results:**

Among the 2,170 participants over the age of 18, the prevalence of adherence to social distancing was 18.5%. Adherence was significantly higher among women; older adults; single, divorced, or widowed people with a low educational level; people of lower socioeconomic status; and people affected by multiple chronic diseases.

**Conclusion:**

Approximately one in five respondents adhered to measures of social distancing, and adherence was more prevalent among the risk groups.

## In Brief

Evaluating the adherence to individual and collective preventive measures against COVID-19 can assist in formulating and improving public health policies aimed at addressing future global health challenges.

## Highlights

■ Approximately one in five respondents adhered to measures of social distancing.
■ Adherence to social distancing was more prevalent in women, older adults with low educational levels, and those affected by multiple chronic diseases.
■ Less than 5% of the population left home normally, whereas the vast majority only went out to carry out essential activities.

## Graphical abstract

**Figure:**
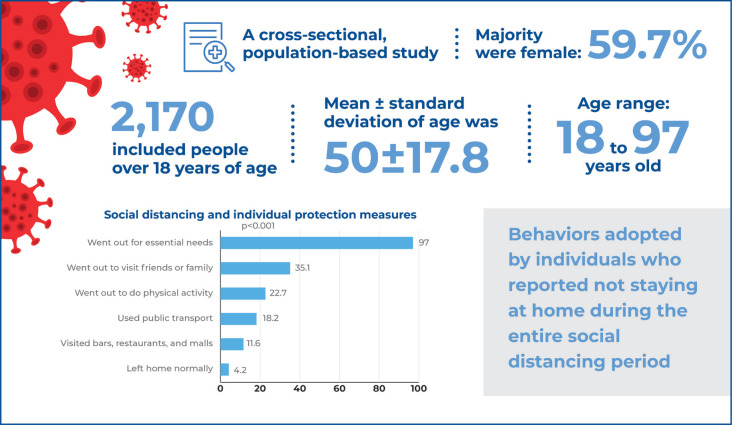

## INTRODUCTION

In May 2023, the World Health Organization declared the end of the Public Health Emergency of International Concern related to Coronavirus Disease 2019 (COVID-19).^(1)^ Three years after the first reported case, 6,951,919 deaths have been recorded worldwide, with 705,054 deaths occurring in Brazil.^(2)^ The high viral transmissibility, slow process of immunization of the population, increasing numbers of individuals with severe respiratory infection, and consequent need for beds in intensive care units help explain both the exponential increase in the number of deaths worldwide, as well as the recommendations for non-pharmacological measures to prevent and mitigate the spread of COVID-19 in the first year of the pandemic.^(3)^

With individual, environmental, and community coverage,^(4)^ the most commonly indicated non-pharmacological measures adopted during the COVID-19 pandemic were the use of face masks, hand hygiene, the use of 70% alcohol, and social distancing.^(5–8)^ This latter is defined as an intervention that aims to reduce or interrupt the chain of disease transmission through physical distancing between infected and healthy individuals, in addition to protecting those at risk of developing severe forms of the disease.^(9)^ Social distancing measures include isolating confirmed cases, quarantining individuals purportedly exposed to the virus, and voluntarily refraining from visiting crowded places.^(4)^

The effect of distancing on the reduction in new COVID-19 cases has been evaluated in various national and international studies. In Brazil, a modeling study,^(10)^ with data from March 14, 2020, to May 1, 2020, found a significant negative correlation (Pearson's ρ=-0.825) with respect to the social distancing index and the rate of the number of new cases, which means that as the first increases, the second decreases. A review of 29 international studies published between January 2020 and March 2020, 10 of which were modeling studies, showed that the combination of social distancing with other prevention and control measures, such as travel restrictions and school closures, reduced the occurrence of new cases, transmissions, and deaths from COVID-19.^(11)^

Considering that adherence to such measures at the population level is, together with vaccination, an effective tool to contain the advance of COVID-19, few studies have investigated adherence to social distancing in Brazil. Understanding the factors that influence adherence to individual protection measures can assist policymakers and healthcare professionals in enhancing communication and awareness strategies in similar crisis scenarios.

## OBJECTIVE

To analyze the frequency of adherence to social distancing and individual protection measures adopted by adult and older populations of two municipalities in southern Brazil and to characterize the sociodemographic aspects of these individuals.

## METHODS

This was a cross-sectional, population-based study that formed part of a larger project entitled "Mental COVID," the objective of which was to assess the impact of COVID-19 on the mental health of the population of the Brazilian municipalities of Rio Grande and Criciúma. Rio Grande^(12)^ is located in the extreme south of Rio Grande do Sul, with an estimated population of 212,000 inhabitants, a human development index (HDI) of 0.744, and a population density of 72.79 inhabitants per km^2^. Criciúma^(13)^ is located in the southern region of Santa Catarina and has approximately 217,000 inhabitants, an HDI of 0.788, and a population density of 815.87 inhabitants per km^2^. The study was conducted during the COVID-19 pandemic, when both municipalities were under social distancing measures.^(14,15)^

The study included adults aged 18 years or older living in private households in urban areas of both cities. The sample size was calculated considering an alpha error of 5%, a statistical power of 80%, a margin of error of 3.0 percentage points, and the addition of a value of 10-20% to control for confounding factors. The representativeness of the target population was ensured using random sampling in two stages. In the first stage, the census sectors of the urban areas of both municipalities were selected according to the 2010 IBGE Demographic Census methodology.^(16)^ The sectors containing private households in urban areas were considered eligible for sampling. In the second stage of selection, 10 households were randomly selected per sector, with the expectation of finding, on average, two adults over 18 years of age in each household. In Criciúma, 60 census tracts were sampled, resulting in 15,765 households, 607 of which were included in the study. In Rio Grande, 90 census tracts were sampled, resulting in 29,734 households, of which 900 were included. All adults living in the selected households were invited to participate, resulting in 2,894 eligible individuals.

As inclusion criteria, an age equal to or greater than 18 years and agreeing to participate in the research were considered. Individuals under 18 years of age and those with conditions that prevented them from adequately responding to the questionnaire at the time of the interview, such as those with cognitive or mental disabilities or conditions that affect lucidity, such as individuals under the influence of narcotics, were excluded. These criteria were identified by either the interviewer or a household resident.

The outcome of interest was the self-reported frequency of adherence to social distancing, evaluated through the following question: "During the period of social distancing, when only essential services were open, did you leave home?",^(17)^ with a dichotomous answer option (no/yes). Negative answers were used as indicators of social distancing adherence. It is important to note that the question was prepared without determining the time period (days or months), as municipal social distancing decrees varied throughout the pandemic in terms of their rules and validity.

The exposure variables were as follows: demographic-sex (male/female), age group (18 to 39/40, to 59/60 or older), skin color (white/other), marital status (married/single, separated, widowed), schooling (elementary/secondary/higher education), socioeconomic status-asset index^(18)^ (lower/intermediate/higher socioeconomic level), health plan (no/yes), and chronic comorbidities. The number of chronic morbidities was assessed by counting the incidence of hypertension, diabetes, heart disease, and obesity. All morbidities had the same weight in the analyses and were obtained through the self-reporting of a medical diagnosis.

The behaviors adopted by those who reported not staying at home during the entire social distancing period were evaluated using the following variables: going out for essential activities (work, supermarket, pharmacy) (no/yes); going out to visit friends or relatives (no/yes); going out to do physical activities (no/yes); using buses at maximum capacity (no/yes); going to bars, restaurants, and shopping malls (no/yes); and leaving home normally (no/yes).

The individual protection behaviors of the participants were analyzed using the following dichotomous independent variables (no/yes): interacting remotely with friends or family, receiving visits from friends or family at home, continuing to work normally, using alcohol during the pandemic, having contact with someone infected with COVID-19, using teletriage and telemedicine services, and being positive for COVID-19.

Data collection took place face-to-face from October 2020 to January 2021, with trained interviewers wearing a jacket, apron, mask, and face shield, as well as using 70% alcohol. The interviews were conducted in front of the participants’ homes using a structured questionnaire with pre-coded questions. Tablets loaded with RedCap^®^ software were used, and the collected data were subsequently transferred to a computer.

Descriptive analyses of the variables were performed using absolute and relative frequencies. The associations between the outcome variable (social distancing) and exposure variables were assessed using Fisher's exact test, with a significance level of 5%. To test the association between demographic and socioeconomic variables and the frequency of distancing, Poisson regression was used, considering the effect of the sample design. The adjustment was made at a single level; that is, all variables were controlled for each other. All analyses were performed using Stata 12.1 statistical package (StataCorp LP, College Station, TX, USA).

This study was approved by the Research Ethics Committee of the *Universidade Federal do Rio Grande*, CAAE: 30955120.0.0000.5324; #4,162,424, in May 2020. All ethical principles recommended by the National Health Council in Resolution no. 466/12 were respected, and all participants authorized the interview through the Free and Informed Consent Registry (RCLE) at the time of the interview.

## RESULTS

Table 1 summarizes the characteristics of the population. Of the 2,894 eligible individuals, 2,170 agreed to participate in the survey, with a response rate of 74%. The majority were female (59.7%), and the average age (mean ± standard deviation) was 50±17.8, ranging between 18 and 97 years.

**Table 1:** Characteristics of the urban populations of the municipalities of Rio Grande/RS and Crisciúma/SC, Brazil, 2020 (n=2.170)

Variable		Sample	Social distancing	Crude analysis	Adjusted analysis†
		n	%	PR (95%CI)	PR (95%CI)
Sex					
	Male	875	12.6	1.00	1.00
	Female	1,295	22.6	1.79 (1.47; 2.20)	1.75 (1.43; 2.15)*
Age group					
	18–39	729	11.0	1.00	1.00
	40–59	763	12.3	1.12 (0.85; 1.49)	1.01 (0.75; 1.36)
	60 or over	678	33.6	3.06 (2.42; 3.87)	2.29 (1.76; 2.99)*
Skin color					
	White	1,815	18.4	1.00	1.00
	Other	347	19.0	1.03 (0.82; 1.32)	0.98 (0.77; 1.24)
Marital status					
	Married	1,066	16.7	1.00	1.00
	Single, separated, widowed	1,104	20.3	1.22 (1.02; 1.45)	1.15 (0.96; 1.39)
Schooling					
	Elementary	921	28.6	2.94 (2.23; 3.86)	2.08 (1.51; 2.86)*
	Secondary	692	12.1	1.25 (0.90; 1.72)	1.19 (0.85; 1.68)
	Higher	555	9.7	1.00	1.00
Asset index (tertiles)					
	1 (lowest)	719	22.8	1.72 (1.36; 2.18)	1.06 (0.83; 1.36)
	2	673	18.4	1.39 (1.09; 1.79)	1.21 (0.95; 1.54)
	3 (highest)	680	13.2	1.00	1.00
Health plan					
	No	1,322	20.2	1.27 (1.05; 1.53)	1.10 (0.90; 1.33)
	Yes	848	15.9	1.00	1.00
Chronic comorbidities					
	0	1.055	12.4	1.00	1.00
	1	544	17.5	1.41 (1.10; 1.79)	1.11 (0.86; 1.44)
	2	263	25.5	2.05 (1.58; 2.67)	1.31 (0.98; 1.74)
	3	97	36.1	2.91 (2.13; 3.96)	1.85 (1.33; 2.58)*
	4	24	45.8	3.69 (2.32; 5.87)	1.74 (1.14; 2.67)*
Social distancing, n (%)			402 (18.5)		

† Poisson regression;

* Statistically significant difference.

PR: prevalence ratio.

The prevalence of social distancing was 18.5% (95%CI= 16.3; 20.8) and was higher in females (22.6%); individuals aged 60 years or older (33.6%); single, separated, or widowed individuals (20.3%); those with less schooling (28.6%); those with a lower socioeconomic level (22.8%); and those without health insurance (20.2%). The following variables remained associated with a greater probability of adhering to distancing: female sex (RR= 1.75; 95%CI= 1.43; 2.15), individuals aged 60 or older (RR= 2.29; 95%CI= 1.76; 2.99), and those with lower education levels (RR= 2.08; 95%CI= 1.51; 2.86). It was observed that the greater the number of chronic conditions, the greater the prevalence of social distancing, ranging from 12.4% in individuals with no morbidities to 45.8% among those with the four morbidities analyzed (hypertension, diabetes, heart disease, and obesity), with a statistically significant difference. The frequency of adherence to social distancing is presented in aggregate form, as the difference between the municipalities of Rio Grande/RS and Criciúma/SC was one percentage point (Table 1).

**Figure 1:**
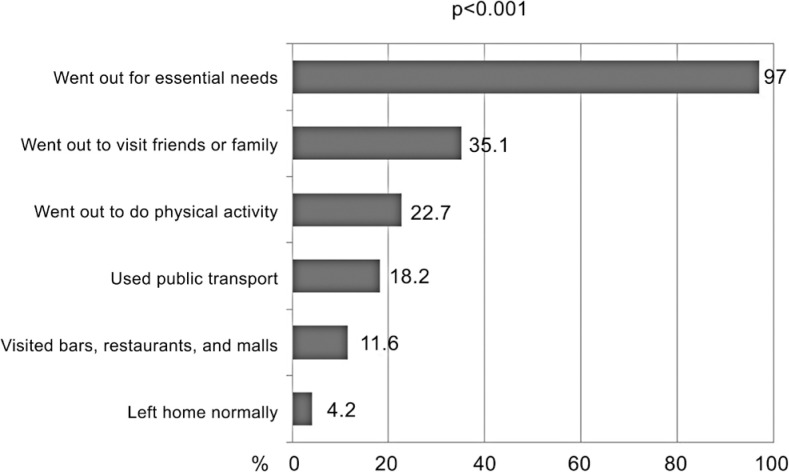
Behaviors adopted by individuals who reported not staying at home during the entire social distancing period. Rio Grande/RS and Crisciúma/SC, Brazil, 2020 (n=1,798)

Among the behaviors adopted by individuals who reported not staying at home during the social distancing period, 97.0% reported going out to perform essential activities, such as working or going to the supermarket and pharmacy, 35.1% went to visit friends and relatives, and 22.7% went out to practice physical activity. Only 4.2% of respondents reported leaving home normally (Figure 1).

**Figure 2:**
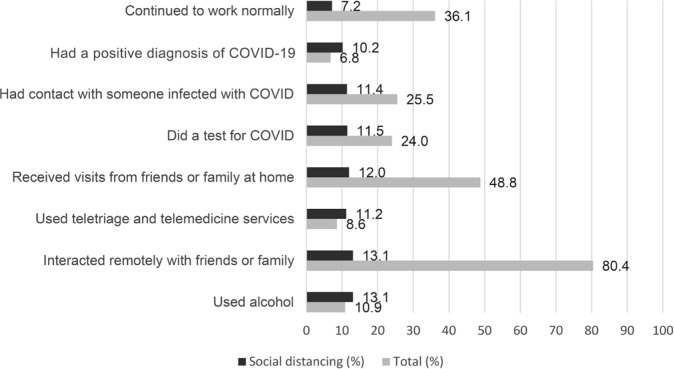
Description of the behaviors of individuals who left home during the social distancing period. Rio Grande/RS and Crisciúma/SC, Brazil, 2020 (n=2.170)

Figure 2 presents the distribution of social distancing according to the behaviors adopted during the pandemic. It was observed that 36.1% of the individuals interviewed continued to work normally during the pandemic, while 7.2% followed recommendations related to social distancing. A positive diagnosis of COVID-19 was reported by 6.8% of the participants, and 10.2% adopted social distancing measures. Among individuals who had contact with someone infected with COVID-19, 11.4% reported adherence to social distancing measures. A test for COVID-19 was reported by 24.0% of the participants, with 11.5% adopting social distancing measures. Among the individuals who reported having received visits from family and friends at home, 12.0% adhered to social distancing. The use of telescreening and telemedicine services, remote interaction with friends and family, and alcohol consumption were reported by 8.6%, 80.4%, and 11.0% of the participants, respectively, with 11.2%, 13.1%, and 13.0%, respectively, following the recommendations related to social distancing.

## DISCUSSION

The frequency of adherence to social distancing was low among the municipalities analyzed. However, among individuals with multiple chronic diseases, the adherence proportion reached almost 50%. Females, individuals aged 60 years or older, and those with less education reported greater adherence to social distancing.

At the international level, a study conducted between March and April 2020 that analyzed adherence to social distancing recommendations among adults in North America and Europe found that 66.9% of respondents reported avoiding leaving their houses at all times, except when accessing essential services.^(19)^ In Brazil, a nationwide study carried out in April 2020 found that 32.0% of respondents were in total isolation; that is, they did not leave their homes.^(20)^

It is important to mention that there were differences in the definitions of social distancing in the data collection periods and in the social distancing guidelines adopted between countries/regions/municipalities, which may justify the variations in the frequencies of social distancing found in the identified studies, making further comparisons difficult. Three hypotheses were proposed to explain the low frequency of adherence to social distancing in this study. First, when considering the national literature reviews, there was a greater frequency of individuals who did not leave their homes and adhered to social distancing at the beginning of the pandemic.^(20–22)^ The second hypothesis was that data collection occurred between the first and second waves of the disease in Brazil, which may have led to a feeling of the end of the pandemic and relaxation in relation to adherence to non-pharmacological measures. The third hypothesis considers the fact that the study represents two cities in the southern region with a relatively high HDI.

In line with the results of the present work, a study conducted in nine municipalities in RS^(6)^ on April 11-13 and 25-27, 2020, and another in the state of Ceará^(23)^ on March 19, 2020, also found that women, older adults, and individuals with less schooling reported greater adherence to social distancing.

These results can be explained as follows. Among females, greater concern and involvement with health lead to preventive behaviors^(24)^ as well as the accumulation, throughout life, of the dichotomy of gender roles and power relations in society.^(19)^ The greater adherence to social distancing by older adults can be explained by retirement, the greater susceptibility of this group to infectious diseases, and the presence of multiple morbidities.^(25)^ The association between low socioeconomic status and education level can be explained by an increase in unemployment in the country during the pandemic. In addition, as these individuals do not have the option to move to safer environments with respect to viral transmission, they reinforce healthcare by staying at home.^(6,19,26)^

The highest proportion of adherence to social distancing among those with the highest number of chronic morbidities was also observed in another Brazilian study of 6,149 individuals over 50 years of age.^(26)^ These results were expected, as this population has the highest number of chronic diseases, with hypertension, diabetes, heart disease, and obesity being the main risk factors for COVID-19. However, the coexistence of these factors increases the risk of disease progression, as it can lead to the intensification of inflammatory processes and a worse prognosis.^(27)^ It is worth noting that multimorbidity is a public health problem in the country and one of the main risk factors for worsening COVID-19. In addition, a study by Xu et al.^(28)^ showed that among patients hospitalized in intensive care units for COVID-19, 72% had previous chronic diseases compared to patients who did not need intensive care.

It was also observed that not all individuals were able to stay at home during the period of social distancing. The behaviors adopted by these individuals were similar to those observed in studies carried out in Quebec,^(26)^ Canada, between April 7 and 15, 2020; in Brazil^(20)^ between April 6 and 8; and in Rio Grande do Sul,^(6)^ Brazil on April 11-13 and 25-27, 2020.

The results showed that less than 5% of the population left home normally, while the vast majority only went out to carry out essential activities, such as working and going to the supermarket and pharmacy. A low frequency of individuals was also observed in places with a greater chance of COVID-19 transmission, such as bars, restaurants, and shopping malls. Furthermore, although public transport is essential for the mobility of individuals with low socioeconomic status, few used it during this period. Regarding physical activity, only one in two respondents reported leaving home to exercise, even though this practice is linked to several health benefits,^(29,30)^ which are even more necessary for the maintenance and rehabilitation of health during a pandemic.

Regarding the behaviors adopted by individuals during the social distancing period, it was observed that staying at home and adhering to social distancing measures were only possible for some groups due to the adoption of teleworking, the closure of schools and universities, cancelation of public events, and approval of emergency aid payments.^(6–8,20)^ Unemployment may also influence adherence to these measures.

As they were less exposed, individuals who stayed at home reported a lower frequency of contact with someone infected with COVID-19, performing diagnostic tests using teletriage and telemedicine services, and having a positive diagnosis of COVID-19.

National and international studies have demonstrated the effectiveness of non-pharmacological protection measures, including the adoption of social distancing, in reducing the transmission of new coronavirus strains and mitigating the impact of the pandemic.^(11,31,32)^ Notably, however, despite less exposure, 10.2% reported a positive diagnosis in our study. Considering that the transmission of coronavirus occurs from person-to-person (symptomatic or asymptomatic) through the air^(33)^ and by personal contact with contaminated saliva droplets,^(34)^ it is possible that adherence to social distancing alone is not sufficient to eliminate the chance of contamination, as these individuals may have had direct contact with infected family, friends, or service providers who transmitted the disease.

Telescreening and telemedicine services were used by 25.5% of the participants, 11.2% of whom adhered to social distancing. The expansion of telehealth in Brazil during the pandemic strengthened the Unified Health System and represented an effective alternative in the fight against coronavirus, as it allowed consultations and monitoring of users to be performed remotely.^(35,36)^ Different studies have demonstrated the effectiveness of telehealth during the COVID-19 pandemic and highlighted the need for greater investment and training of health professionals to use this technology during and after health crises.^(36)^

Alcohol use during the pandemic was reported by 13.0% of individuals who adhered to social distancing. This prevalence was lower than that observed in other studies,^(37,38)^ which found that alcohol consumption was associated with fear of dying or losing a loved one, sadness, depression, anxiety, and insecurity about employment.^(37,38)^ It is worth highlighting that unemployment, the adoption of home office work, and less access to places of consumption may have influenced the reduction in the level of alcohol consumption and explain the results obtained.^(39)^

This study has some limitations that should be considered. First, the frequency of adherence to social distancing may be subject to memory bias since data collection was carried out over three months. In addition, responses to questions about adherence to social distancing and preventive measures may have been overestimated among participants who had the disease, those with greater fear or concern about being contaminated, and those who felt embarrassment about reporting poor adherence.

Among its strengths, this is one of the few Brazilian population-based studies that guaranteed a representative sample of the population of Rio Grande, RS, and Criciúma, SC. In addition, as it was conducted in individuals’ homes, the study included those who did not have access to the Internet. Therefore, the results may be applicable to other regions of the country and contribute to future awareness campaigns, educational programs, and social support strategies during health crises.

## CONCLUSION

Therefore, it can be concluded that approximately one in five individuals adhered to social distancing, with greater adherence among the risk groups. The majority of individuals adopted protective measures, such as mask use, hand washing, and 70% alcohol use, and more than half interacted remotely with friends or family, which may have contributed to the reduced number of COVID-19 cases in this group.

This study contributes information about the social and behavioral characteristics of people who did or did not adhere to preventive measures against COVID-19, which can serve as a reference for health services and government entities in future public health emergencies, helping to reduce the burden on healthcare services. However, new longitudinal modeling studies are necessary to minimize temporality errors and improve conclusions regarding individuals’ adherence to preventive measures.
